# Tribute to the Memory of Dr. W. D. Bizzell

**Published:** 1890-08

**Authors:** 


### TRIBUTE TO THE MEMORY OF DR. W. D. BIZZELL.

At a meeting of the Atlanta Society of Medicine, held July 1st, 1890, the following resolutions were unanimously adopted:

WHEREAS, Our esteemed and beloved fellow-member, Dr. W. D. Bizzell, has been removed from among us by the hand of death; therefore be it

*Resolved*, That in the death of Dr. Bizzell this Society has lost, not only an active and zealous member, and one who always strove, by word and deed, to promote its interests, but also a true-hearted and generous friend, who, in all the relations of life, fulfilled with earnestness of purpose and with kindness of heart, every social obligation which was imposed upon him in his contact with his fellow men.

That this Society extends its heartfelt sympathy to his bereaved family, who have sustained in his death an irreparable loss.

That these resolutions be published in the daily papers and medical journals of this city, and that a copy be furnished to the family of the deceased.

---

The degree of LL. D. has been conferred on Dr. Robert Battey of Rome, Ga., by the Jefferson Medical College.

---

Dr. A. W. Calhoun of Atlanta has received the degree of LL. D. from the University of Georgia.

